# The Prevalence of Domestic Verbal Abuse in Pregnant Women in a Semi-urban Area

**DOI:** 10.7759/cureus.60740

**Published:** 2024-05-21

**Authors:** Pankaj Salvi, Sneha Aramandla, Vidya Gaikwad, Swati Ghonge

**Affiliations:** 1 Obstetrics and Gynecology, Dr. D. Y. Patil Medical College, Hospital and Research Centre, Dr. D. Y. Patil Vidyapeeth (Deemed to be University), Pune, IND; 2 Community Medicine, Dr. D. Y. Patil Medical College, Hospital and Research Centre, Dr. D. Y. Patil Vidyapeeth (Deemed to be University), Pune, IND

**Keywords:** fetal outcome, domestic violence, verbal abuse, semi-urban, maternal outcome

## Abstract

Introduction

Domestic violence (DV) in the form of verbal abuse is very common among women, especially pregnant women, posing as a serious public health issue that could lead to complications in pregnancy and threaten maternal and fetal outcomes. Studies have determined that domestic verbal abuse (DVA) in pregnancy was more common in women less than 25 years of age as well as in those with low education levels. This study determined the overall prevalence of verbal abuse in pregnant women, in a semi-urban population and is unique in that the verbal abuse in pregnant women with a previous girl child was also determined. This study helps healthcare providers identify the potential causes of DVA in pregnancy and provide timely interventions in the form of counseling for pregnant women and families.

Objective

This observational study was carried out to assess the prevalence of DVA among pregnant women, to determine the trimester of occurrence of DVA among pregnant women, and to explore the associations of DVA with age, employment status, parity gestational age, and birth weight.

Materials and methods

This was a six-month hospital-based observational study conducted at Dr D. Y. Patil Medical College's in-patient department (IPD) of Obstetrics and Gynecology in Pimpri, Pune. Consent was obtained from 200 pregnant women who received admission for delivery and provided a validated modified copy of a DV assessment screening questionnaire. A statistical analysis was performed using GraphPad Prism 10. A Chi-square test was employed wherever required, and a p-value of less than 0.05 was considered significant.

Results

The study included 200 pregnant women, who were admitted to the hospital for delivery. The prevalence of DV in the form of verbal abuse was noted to be 74 out of 200 (37%). The working status of the woman showed an influence on DVA. There was also a significant increase in verbal abuse (68%) among the age group between 18 and 23 years. The previous delivery of a female child also had a significant impact on DVA, which turned out to be more prevalent, particularly if two female children were born previously (80%). The study also noted higher rates of preterm deliveries in pregnant women with DVA being 57%.

Conclusion

The study demonstrates that women, even in modern times, experience DVA during pregnancy, especially among the younger age group. It has also been found that it is more common among women who are financially dependent due to maternal unemployment. As a result, there is a need to routinely screen pregnant women for DVA to avoid potentially detrimental pregnancy outcomes and to prevent ongoing abuse.

## Introduction

The United Nations defines violence against women as "any act of gender-based violence that results in, or is likely to result in, physical, sexual, or mental harm or suffering to women, including threats of such acts, coercion, or arbitrary deprivation of liberty, whether occurring in public or private life” [1]. Women are frequent targets of domestic abuse, with data indicating that it constitutes approximately 5% of the healthy years lost by women in nations with poor economies [2]. Domestic abuse, commonly known as domestic violence (DV), including domestic verbal abuse (DVA), refers to a pattern of behavior that is employed in any relationship to establish or maintain power and control over a spouse or partner. This encompasses any action that alarms, intimidates, terrorizes, manipulates, harms, degrades, blames, or hurts someone [3]. DVA is a variety of phrases or actions intended to coerce, threaten, and uphold authority and control over someone. These consist of taunts, acts of shame and mockery, silent treatment, and attempts to frighten and isolate [4].

DVA in pregnancy frequently goes undetected because victims intend to keep it a secret due to sociocultural norms or are concerned about consequences with their abuser(s) in the event they expose the occurrence [2]. Several factors have been attributed to the prevalence of domestic abuse during pregnancy in some households. These variables include either spouse's low educational status, no or low employment position, impoverishment, unhygienic living conditions, the absence of a male offspring, religious differences, a lack of medical treatment, alcoholism, financial restrictions, and so on [2].

Violence during pregnancy puts at risk both the mother and the baby's health, resulting in a variety of complications such as preterm labor in women, abortion, premature separation of the placenta, and premature rupture of membranes [5]. DV in pregnancy also creates issues for the infant, including low birth weight, fractured bones, soft tissue injury, rupture of the lung or spleen, and hypoxia in the fetus [6]. Abortions, limited contraceptive use, and unintentional pregnancies are all linked to violence [7].

Studies have shown that the incidence of violence within communities, nations, and districts, or between them, indicates that abuse is avoidable and can be abolished. Therefore, there is a need to assess the prevalence of DV and further education among the population [8].

## Materials and methods

Study design and setting

This was a hospital-based observational study performed at Dr. D. Y. Patil Medical College, Hospital and Research Centre. This study was reviewed and approved by the DY Patil University Institutional Ethics Subcommittee, with reference number: I.E.S.C./25/2023.

Participants

All pregnant women in the age group of 19-40 years who were admitted to the hospital for delivery between August 1 and February 1, 2023, and who were willing to participate in the study were included. Women with known mental illnesses were excluded. 

Inclusion and exclusion criteria

The inclusion criteria for the study comprised women aged between 19 and 40 years who delivered at the institute and were willing to participate. Pregnant women with pre-existing mental illnesses were excluded from the study.

Sample size

A validated modified version of the Abuse Assessment Screen questionnaire was used by a single interviewer on 200 pregnant women admitted for delivery after obtaining their consent.

Data collection and consent

We collected the data using a preformed case record form and a validated modified version of an Abuse Assessment Screening questionnaire, predesigned, pretested, and developed by Neil Jacobson and John Gottman [9]. This questionnaire was used by an interviewer on 200 pregnant women admitted for delivery (Table 9). Each woman was interviewed separately by a single interviewer on the bedside, after feeding her baby, to provide a comfortable and calm environment. Written and informed consent was obtained from all participants following a comprehensive discussion with the patient, ensuring respect for their privacy.

Statistical analysis

Data entry was done utilizing MS Excel (Microsoft Corporation, Redmond, Washington, United States). A statistical analysis was performed using GraphPad Prism 10. Relevant descriptive statistics were analyzed and plotted as frequency and percentage. To ascertain significant associations and patterns within the dataset, Chi-square test was applied wherever needed, and a p-value of 0.05 or less was considered significant.

## Results

The study involved 200 pregnant women. The majority of the study population, 86 (43%) pregnant women, were between the ages of 23 and 27 years, subsequently followed by 59 (29.5%) pregnant women between the ages of 28 and 32 years, 41 (20.5%) pregnant women between the ages of 18 and 22, and 14 (7%) pregnant women between the ages of 33 and 38 years (Table 1).

**Table 1:** Age distribution

Age group (years)	Frequency	Percentage
18-22	41	20.5
23-27	86	43
28-32	59	29.5
33-38	14	7
Total	200	100

In our study population, 101 (50.5%) of pregnant women were primigravida, while the remaining 99 (49.5%) were multigravida. The majority of the pregnant women, accounting for 141 (70.5%), had term delivery, while 59 (29.5%) had preterm delivery. We also analyzed the working status of these pregnant women and found that 165 (82.5%) were nonworking and 35 (17.5%) were working (Table 2).

**Table 2:** Demographics of pregnant women

Parameter	Frequency (%)
Primigravida	101 (50.5%)
Multigravida	99 (49.5%)
Preterm delivery	59 (29.5%)
Term delivery	141 (70.5%)
Working	35 (17.5%)
Nonworking	165 (82.5%)

Association of age group with DVA

The association between age group and incidence of DVA in the study population was analyzed using the Chi-square test. It was found that the association was statistically significant (p < 0.0001) (Table 3).

**Table 3:** Association of age group with DVA DVA: Domestic verbal abuse *Significant p-value

Victims of DVA	Age group					
	18-23	24-27	28-32	33-38	Total	p-value
Yes	28 (68%)	18 (21%)	22 (37%)	6 (43%)	74 (37%)	<0.0001*
No	13 (32%)	68 (79%)	37 (63%)	8 (67%)	126 (63%)	
Total	41	86	59	14	200	

Association of the professional status of the patient with DVA

With further interest in looking at whether the working status of the pregnant women in our study had any association with the incidences of DVA, the association was analyzed through a Chi-square test, and it was found that the association was statistically significant (p < 0.0001) (Table 4).

**Table 4:** Association of the professional status of the patient with DVA DVA: Domestic verbal abuse *Significant p-value

Occupation status (patient)	Victims of DVA			
	Yes	No	Total	p-value
Working	2 (6%)	33 (96%)	35	<0.0001*
Nonworking	72 (44%)	93 (56%)	165	
Total	74 (37%)	126 (63%)	200	

Association between the professional status of the pregnant women’s partners and the incidences of DVA

DVA has been closely linked to the partners’ educational and professional status. Therefore, to analyze the association between the professional level of the partners of our patients and the incidences of DVA, the professional level of the partner was divided into three levels. Level I included professions that required primary education or below, level II included professions that required high school education, and level III included professions that require higher education (graduates in professional degree or nonprofessional degree). A Chi-square test was used and found that the association was not statistically significant (p = 0.073) (Table 5). Although it was noted that the prevalence of DVA was higher in partners with level I professional education as compared to the others.

**Table 5:** Association between the professional status of the patients’ partners with the incidences of DVA DVA: Domestic verbal abuse

Professional level (partner)	Victims of DVA			
	Yes	No	Total	p-value
Level I	39	61	100	0.073
Level II	32	47	79	
Level III	3	18	21	
Total	74	126	200	

Association between previous children and DVA

Pregnant women who have had one or more female children previously have been seen to have an additional layer of vulnerability to the traumatic effects of DVA. A Chi-square test was used to analyze the association between the sex of the previous child and the incidence of DVA. It was found that the association was indeed statistically significant (p < 0.05) (Table 6).

**Table 6:** Association between the previous child and DVA DVA: Domestic verbal abuse *Significant p-value

Previous child	Victims of DVA			
	Yes	No	Total	p-value
Only boy	12 (36%)	21 (64%)	33	<0.05*
One girl	16 (34%)	31 (66%)	47	
More than one boy	1 (17%)	5 (83%)	6	
More than one girl	8 (80%)	2 (20%)	10	
One boy one girl	2 (67%)	1 (33%)	3	

Association between DVA and birth weight

DVA during pregnancy can have significant adverse effects on the birth weight of infants. Studies have shown a clear association between exposure to DVA and adverse pregnancy outcomes, including low birth weight. The analysis found that there was a significant association between the incidences of DVA and the fetal birth weight (p < 0.0001) (Table 7).

**Table 7:** Association between DVA and birth weight DVA: Domestic verbal abuse *Significant p-value

Birth weight	Victims of DVA			
	Yes	No	Total	p-value
IUD	1 (50%)	1 (50%)	2 (1%)	<0.0001*
Low	32 (57%)	24 (43%)	56 (28%)	
Normal	41 (29%)	101 (71%)	142 (71%)	
Total	74	126	200	

Association between DVA and gestational age

High levels of stress and anxiety can contribute to complications such as preterm birth. Therefore, Chi-square test was used to find the association between DVA and gestational age. However, it was found that the association was not statistically significant (p = 0.4858) (Table 8).

**Table 8:** Association between DVA and gestational age DVA: Domestic verbal abuse

Gestational age at delivery	Victims of DVA			
	Yes	No	Total	p-value
Preterm	24 (41%)	35 (59%)	59 (29.5%)	0.48
Term	50 (35%)	91 (65%)	141 (70.5%)	
Total	74 (37%)	126 (63%)	200	

Insights into the ultrasound findings among pregnant women with DVA

**Figure 1:**
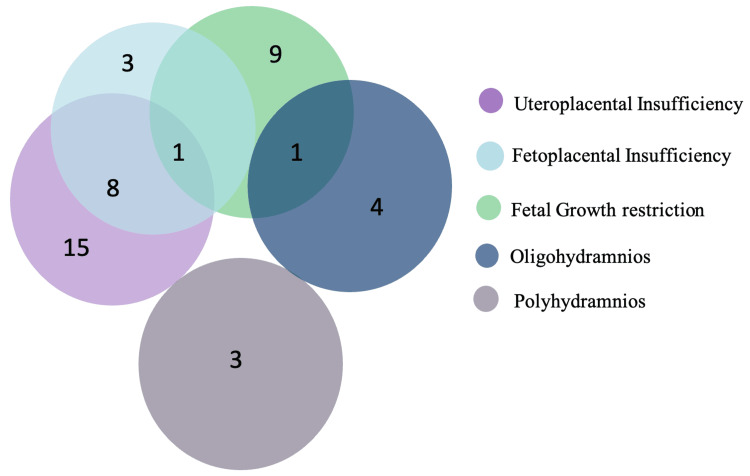
Venn diagram showing ultrasound results among the study population

As the gestational age at delivery is an outcome of several underlying factors, we analyzed the ultrasound results and found that the majority, accounting for 15 (7.5%) of the pregnant women had uteroplacental insufficiency, fetal growth restriction in nine (4.5%), oligohydramnios in four (2%) pregnant women, and polyhydramnios in three (1.5%) pregnant women each. Some pregnant women had more than one condition, as indicated in the Venn diagram (Figure 1).

## Discussion

The primary goal of this study was to determine the prevalence of DVA among pregnant women as well as the factors that influence it. According to the findings of this study, verbal abuse was the most common form of DV (37%). Individual, relationship, community, and social concerns all contribute to the risk of domestic and family violence. There is a negative correlation between education and DVA. Lower education levels are associated with a higher risk of DVA [10]. The relationship between women's education and spousal abuse varied by community, with evidence indicating that communal acceptance of mistreatment reduced higher education's protective effect [11]. Many studies have found that violence is associated with poor pregnancy outcomes. Abused women are far more inclined to delay prenatal treatment, experience early labor, have miscarriages, or have low-birth-weight babies [8]. Continuous stress appears to influence prenatal outcomes through changes in individual behavior, interfering with their ability to sustain adequate nutritional status, rest, healthcare, or adjust physiological responses [8]. The current study found a significant rise in DVA among those aged 18-23 years (68%). A study by Samal et al. found a 7.9% prevalence of DVA among individuals under the age of 25 [12]. Another study by Lin et al. found that those exposed to DV had significantly shorter mother ages (p < 0.001) [13]. According to the current study, there is a 44% higher incidence of DVA among pregnant women who are not working. In a study by Samal et al., it was reported that women who completed high school or higher had a reduced incidence of DVA (4.8%) [12]. Another study by Calikoglu et al. found that better educational status reduced the incidence of DV by 12.8 times [14]. DV during gestation was also twice as prevalent among women with less than a high school education (OR = 2.0; 95% CI: 1.17-3.25) as well as those who did not have employment (OR = 2.0; 95% CI: 1.27-3.19) [2].

According to the current study, there is a 41% relationship between DV during pregnancy and low-birth-weight births among pregnant women. In a study conducted by Lin et al., the percentage of low birth weight in the DV-exposed group was significantly higher compared to the unexposed group during full-term childbirth (4.9% vs. 3.3%, p = 0.001) [13].

In India, it is common knowledge historically that there is a relevance to having a girl child, who is treated as a burden. In this study, a unique aspect was added to note the prevalence of DVA in pregnant women who had given birth to a girl child previously. This study found that the previous delivery of a female child had a significant impact on DVA, which was more common, particularly if two female children were born prior (80%). Mahapatro et al. discovered that the desire for male children was much higher (24%) in women experiencing DVA, whereas the demand appeared almost negligible when such an incidence was not observed, which was also statistically significant (z = 6.519; p = 0.0001) [15].

The current study found that partner education had a 39% impact on DVA during pregnancy. Emotional or psychological abuse is the least examined, with few studies on its associated components, because most women do not see it as a kind of abuse and do not openly accept it [12]. The repercussions of DVA on young children during pregnancy continue after delivery. Women who suffer DV during their pregnancy or in the year before pregnancy are much less likely to nurse their newborns, and those who do start nursing are more likely to stop by four weeks postpartum [16]. 

The American College of Obstetricians and Gynecologists (ACOG) suggests that all women be evaluated for signs and symptoms of DV during routine and prenatal visits [10]. Pregnancy is the one time where healthy women have frequent interaction with health care professionals, which may allow doctors to spot abuse more quickly [17]. Providers should provide assistance and referral information. 

Pregnant women are more likely to experience DV than to develop preeclampsia or gestational diabetes. DV is especially dangerous since it threatens both the mother and the fetus. Healthcare practitioners should understand the psychosocial effects of DVA during pregnancy [10]. If medical professionals fail to respond to the woman, it can lead to revictimization and exacerbate feelings of self-pity, hopelessness, and discrimination [18].

Limitations of the study

This study was limited to the semi-urban population admitted for delivery, while there should be a larger study at the community level to find out the prevalence of DVA, its associated factors, and measures to safeguard women.

## Conclusions

This research discusses the critical issue of DVA among pregnant women and its profound implications for maternal and fetal health. The results underscore the need for increased awareness strategies to reduce such incidences. As our results suggest, DVA during pregnancy is not only associated with the immediate risks to the well-being of expectant mothers but also with the growth and development of the unborn child. It is therefore of critical importance in the interest of public health that healthcare professionals, policymakers, and advocacy groups collaborate to elucidate the root causes of DVA, provide support and resources for affected women, and implement strategies that encompass education and empowerment of pregnant women.

## Appendices

**Table 9:** Questionnaire

Questions	True	False
(1) Insults	True	False
(2) Name-calling	True	False
(3) Accusations on your character or conduct etc	True	False
(4) Insults for not having a male child	True	False
(5) Insults for not bringing dowry etc	True	False
(6) Preventing you or a child in your custody from attending school, college or any other educational institution	True	False
(7) Preventing you from taking up a job	True	False
(8) Forcing you to leave your job	True	False
(9) Preventing you or a child in your custody from leaving the house	True	False
(10) Preventing you from meeting any person in the normal course of events	True	False
(11) Threat to commit suicide	True	False
(12) Any other verbal or emotional abuse	True	False
(13) I have to do things to avoid my partner’s jealousy	True	False
(14) My partner tries to control who I spend my time with.	True	False
(15) My partner repeatedly accuses me of flirting with other people.	True	False
(16) My partner is overly suspicious that I am unfaithful.	True	False
(17) My partner keeps me from going places I want to go.	True	False
(18) My partner forcibly tries to restrict my movements.	True	False
(19) My partner tries to control all my freedom.	True	False
(20) My partner tries to convince other people that I’m crazy.	True	False
(21) My partner insults my family.	True	False
(22) My partner humiliates me in front of others.	True	False
(23) My partner makes me do degrading things.	True	False
(24) My partner intentionally does things to scare me.	True	False
(25) My partner threatens me physically during arguments.	True	False
(26) My partner warns me that if I keep doing something, violence will follow.	True	False
(27) My partner threatens to hurt someone I care about.	True	False
(28) My partner intentionally damages things I care about.	True	False
(29) My partner does cruel things to pets or other animals.	True	False
(30) My partner threatens to hurt my children.	True	False
